# Potential of Flavonoid-Inspired Phytomedicines against COVID-19

**DOI:** 10.3390/molecules25112707

**Published:** 2020-06-11

**Authors:** Wilfred Ngwa, Rajiv Kumar, Daryl Thompson, William Lyerly, Roscoe Moore, Terry-Elinor Reid, Henry Lowe, Ngeh Toyang

**Affiliations:** 1 Brigham and Women’s Hospital, Harvard Medical School, Boston, MA 02115, USA; 2 Dana-Farber Cancer Institute, Harvard Medical School, Boston, MA 02115, USA; 3 Northeastern University, Boston, MA 02115, USA; rajivtondak@gmail.com; 4 R&D Biomedical Materials, Millipore Sigma, Milwaukee, WI 02115, USA; 5 Global Research and Discovery Group Sciences, Winter Haven, FL 33884, USA; d.thompson@globalrdg.com (D.T.); william.lyerly@gmail.com (W.L.); rmoore@phrockwood.com (R.M.); 6 School of Pharmacy, Concordia University of Wisconsin, Mequon, WI 53097, USA; Terry-Elinor.Reid@cuw.edu; 7 Vilotos Pharmaceuticals Inc, Baltimore, MD 21202, USA; lowebiotech@gmail.com (H.L.); ngeh.toyang@flavocure.com (N.T.); 8 Flavocure Biotech Inc, Baltimore, MD 21202, USA

**Keywords:** flavonoids and their derivatives, phytomedicine, COVID-19, SARS-COV-2, smart nanoparticles

## Abstract

Flavonoids are widely used as phytomedicines. Here, we report on flavonoid phytomedicines with potential for development into prophylactics or therapeutics against coronavirus disease 2019 (COVID-19). These flavonoid-based phytomedicines include: caflanone, Equivir, hesperetin, myricetin, and Linebacker. Our in silico studies show that these flavonoid-based molecules can bind with high affinity to the spike protein, helicase, and protease sites on the ACE2 receptor used by the severe acute respiratory syndrome coronavirus 2 to infect cells and cause COVID-19. Meanwhile, in vitro studies show potential of caflanone to inhibit virus entry factors including, ABL-2, cathepsin L, cytokines (IL-1β, IL-6, IL-8, Mip-1α, TNF-α), and PI4Kiiiβ as well as AXL-2, which facilitates mother-to-fetus transmission of coronavirus. The potential for the use of smart drug delivery technologies like nanoparticle drones loaded with these phytomedicines to overcome bioavailability limitations and improve therapeutic efficacy are discussed.

## 1. Introduction

The 2019 outbreak of the novel coronavirus disease (COVID-19) caused by severe acute respiratory syndrome coronavirus 2 (SARS-CoV-2) catapulted governments and scientists in a race to find a vaccine or drug for the prevention or treatment of this deadly pandemic. As scientists scramble to find effective therapies, doctors are trying cocktails of unproven therapies and desperate populations are trying different phytomedicines [1]. Many clinical trials to test different drugs and phytomedicines for COVID 19 have been launched [2].

Phytomedicine, the use of medicinal plants for prevention and treatment of disease, is of growing importance worldwide [3], but has been largely absent in global health as in the case of COVID-19. A number of studies have surfaced on the use of Chinese traditional medicines including phytomedicines on the treatment of COVID-19 and there are anecdotal reports on phytomedicines discovered for the treatment of COVID-19 in Africa, particularly in Madagascar [4,5]. Phytomedicine of proven quality, safety, and efficacy, contributes to the World Health Organization (WHO)’s global health priority of ensuring that all people have access to quality healthcare. However, the use of phytomedicine is largely driven by anecdotal evidence, and is limited by poor bio-availability. Cross-disciplinary research collaborations could overcome these barriers and help accelerate development of effective evidence-based phytomedicines for COVID-19, that would also be widely accessible in developing countries where over 80% of the population uses phytomedicine. Flavonoids have been reported to possess various disease prevention and treatment potential including against viruses [6,7]. Here we report new findings on a number of flavonoid phytomedicines with potential for development into COVID-19 therapeutics or prophylactics.

## 2. Results and Discussions

It is now well established that SARS-CoV-2 uses the angiotensin-converting enzyme 2 (ACE2) receptors abundant in the respiratory tract and lungs to infect cells [8,9,10]. CoV-2 spike glycoprotein binds ACE2 cellular receptors to facilitate fusion and ultimately entry into cells. Chloroquine (CLQ) is being investigated as a prophylactic or therapeutic and is reported to inhibit the activity of CoV-2, thus launching the investigation into possible binding sites to ACE2 [11]. Molecular docking studies were employed to investigate different flavonoid molecules’ potential binding efficacy to ACE2 metallopeptidase domain. We are however aware that there are other possible binding sites of these molecules, including the CARS-CoV-2RBD domain of ACE2 [12].

**Figure 1:**
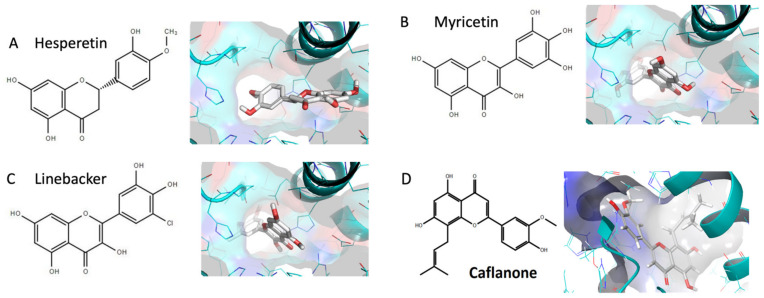
Docking studies for (**A**) Hesperetin; (**B**) Myricetin; (**C**) Linebacker; (**D**) Caflanone (FBL-03G).

**Figure 2:**
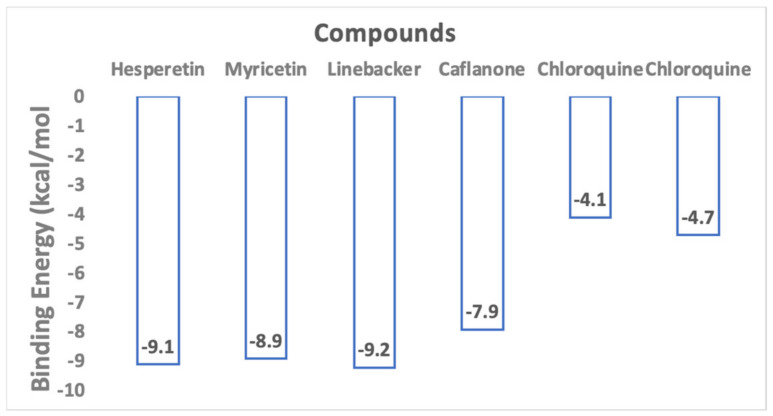
Docking study results show flavonoid molecules can bind very effectively to the ACE2 receptor used by SARS-CoV-2 to infect cells. Two binding energies are shown for chloroquine due to two possible binding poses in different regions of the metallopeptidase domain.

Figure 1 highlights flavonoids investigated by us including hesperetin [7], myricetin [13], Linebacker, and caflanone (FBL-03G) [14]. Binding energy results are highlighted in Figure 2 in comparison to that of CLQ, which is currently in clinical trials as a potential prophylactic and treatment of COVID-19. These preliminary works indicate that flavonoids could bind with high affinity to the spike protein, helicase, and protease sites on the ACE2 receptor causing conformational change to inhibit viral entry of coronaviruses. For comparison, the results indicate that the investigated flavonoids could bind equally or more effectively than CLQ. The binding results highlight potential for considering these flavonoids as prophylactic against SARS-CoV-2.

**Figure 3:**
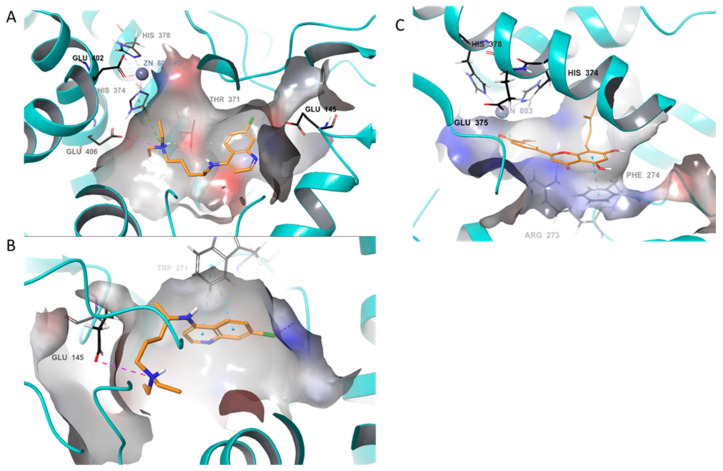
Predicted binding poses of caflanone and chloroquine (CLQ) within the zinc metallopeptidase domain of ACE2 (cyan cartoon and electrostatic potential surface). (**A**,**B**) Two predicted poses of CLQ (orange), one of which interacts with HIS374, which is coordinated with Zn and the other binds further into the cleft of the catalytic domain, away from Zn. (**C**) Caflanone (orange) is predicted to bind within the catalytic domain similar to CLQ in A.

A close look at the interaction of CLQ seen in Figure 3A,B indicates two possible binding poses in different regions of the metallopeptidase domain. Although CLQ seen in Figure 3A exhibits a lower affinity for the site, it is making a key pie-cation interaction with His374 that in turn coordinates with zinc (ZN). The second predicted pose of CLQ inverts the structure allowing for binding deeper into the catalytic site and farther away from zinc. As a result, the chloroquinoline group of CLQ makes pie stacking interactions with the residue of Thr371 and a salt bridge interaction with Glu406 (Figure 3B). The structurally diverse caflanone also interacts within the catalytic site of ACE2 (Figure 3C) and makes multiple favorable interactions with Glu375, Glu402 that coordinates with ZN, Phe274, and Arg273.

Moreover, in vitro study results (Table 1) for caflanone show potential to inhibit virus entry factors including, ABL-2, [15] cathepsin L, [16] cytokines (IL-1β, IL-6, IL-8, Mip-1α, TNF-α), [17], and PI4Kiiiβ [18], as well as AXL-2, which facilitates mother-to-fetus transmission of coronavirus [19]. 

Similarly, Equivir, a blend of natural bioflavonoids: hesperetin, myricetin, and piperine demonstrated antiviral efficacy. Equivir has a synergistic effect by inhibiting ICAM1 (Intercellular adhesion molecule 1), ATPase, helicase, polymerase and neuraminidase to reduce or prevent viral entry, transcription, replication, and budding. It has potential as a treatment and a preventative prophylactic. The components hesperetin, myricetin, and piperine are all FDA listed as Generally Recognized as Safe (GRAS). Other studies [7,13] have reported other flavonoid compounds with preliminary results showing anti-coronavirus and anti-inflammatory activity. Altogether, these results provide significant impetus for further investigation of these phytomedicines in the fight against COVID-19.

Despite the promising in silico and in vitro results, a major limitation of flavonoids in in vivo and clinical translation studies is the poor bioavailability of flavonoids [20]. Thus, there is often need to enhance the bioavailability of these flavonoids in-vivo. To this end, approaches to increase bioavailability include the use of absorption enhancers, improving metabolic stability, changing the site of absorption from large intestine to small intestine, and the use of drug delivery systems [20].

**Figure 4:**
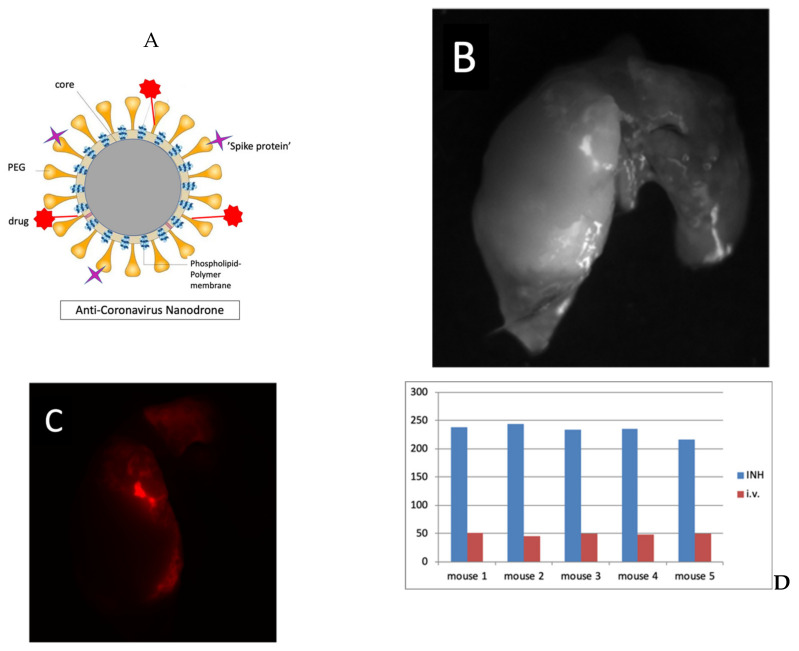
(**A**) A schematic of the anti-COVID-19 (AC) nanodrone designed to target ACE2 receptor allowing for image-guided monitoring of space-time distribution and treatment monitoring; (**B**–**C**) results of smart nanoparticles (RGD-pGNPs) targeting lung lesions; (**D**) Fluorescence intensity of smart nanoparticles targeting lung lesions in mice administered via INH (pulmonary delivery) versus via i.v. (intravenous delivery).

Smart nanoparticles present as viable drug delivery systems. The SARS-CoV-2 itself is the quintessential smart nanoparticle of 60–140 nm [8,19,21], and has together with other viruses been described as smart and capable nanoparticles that cause human loss, havoc, and devastation [22]. One approach to counter this is to also develop anti-COVID-19 (AC) smart nanoparticles (nanodrones). Figure 4A illustrates such an AC nanodrone with the capacity to target the ACE2 receptor and deliver both hydrophobic and hydrophilic payloads. The flavonoids can be encapsulated in the nanodrones using microfluidic approaches [23]. The flavonoids can also be conjugated as targeting moieties on the AC nanodrones. Our preliminary work with nanodrones for targeting lung lesions (Figure 4B–E) suggests that such a pulmonary (INH) route of delivery may be advantageous with higher amounts of payload reaching the lung [24,25,26], which is ground zero for COVID-19 [1]. Here the targeting moieties conjugated on the nanoparticle drones could be flavonoid molecules with targeting SARS-CoV-2 infection in the lungs.

Beside addressing bioavailability, use of AC nanodrones could allow the ability for image-guided targeting and monitoring including via computed tomography (CT) or magnetic resonance imaging (MRI). While nanodrones [23,26,27] can use gold nanoparticles as core material, this could be readily replaced with iron or gadolinium for MRI. Using the latter would allow them to be directed with magnetic fields for superior targeting to disease sites.

In perspective, previous studies on CLQ have shown that chloroquine can block endocytosis of nanoparticles [28]. The docking study results above suggest that smart nanoparticles with flavonoids might be more effective in binding the ACE2 receptor and could represent a significant improvement or advance in targeting such indications. Smart nanoparticles can also be constructed along the lines of the multi-compartment liposome [29], which have hydrophobic and hydrophilic compartments to carry flavonoids or other drugs for more effective targeting to enhance their effectiveness. In general, advantages of using such smart technology includes better disease targeting, ability to cross membranes and enter cells, longer duration drug action, lower doses, and reduced side effects, which is of vital importance.

## 3. Conclusions

In summary, our preliminary results highlight significant potential for COVID-19 phytomedicines in the war against the novel coronavirus pandemic. While the activity of other flavonoids like hesperetin and myricetin are known, the results for flavonoids like caflanone and linebacker are presented as new candidates for further investigation. Overall, the findings justify further cross-disciplinary research collaborations investigating such viable flavonoids and leveraging advances in smart nanomaterials for targeted delivery. This could accelerate the development of effective phytomedicines for COVID-19 that could save innumerable lives, with capacity for expedient adaptation to combat likely future pandemics.

## 4. Materials and Methods

### 4.1. In-Silico Studies

Molecular Docking is an effective and competent tool for in silico screening, which plays an important and ever-increasing role in rational drug design. Molecular modeling approaches are increasingly being used to accelerate the drug discovery process. Molecular docking is an effective molecular modeling tool that is used to identify key interactions of ligands within the active site of a target protein and assigns a predicted binding affinity. Docking was performed using the Schrödinger software suite (Maestro, version 11.8.012, Schrödinger New York, NY, USA)following well-established approaches described in previous studies [13,30,31]. Compounds were extracted from the PubChem database in the SDF format and were combined in one file. The file was then imported into Maestro and prepared for docking using LigPrep. Ioniser was used to generate an ionized state of all compounds at the target pH 7 ± 2. This prepared low-energy conformers of the ligand were taken as the input for an induced—fit docking. Prior to docking, the atomic coordinates of the crystal structure of SARS-CoV were retrieved from the Protein Data Bank (PDB 1R4L). The protein was prepared with the Protein Preparation Wizard where bond orders and hydrogens were assigned, missing side chains and loops were filled, heteroatomic states generated and restrained minimization with OPLS_2005 force field conducted. The induced-fit docking protocol was run from the graphical user interface accessible within Maestro 11.8.012 and Maestro 12.1. Receptor sampling and refinement were performed on residues within 5Å of each ligand for each of the ligand–protein complexes. Induced-fit receptor conformations were generated for each of the ligands. Re-docking was performed with the test ligands into their respective structures that are within 30 kcal/mol of their lowest energy structure. Finally, the ligand poses were scored using GlideScore scoring functions.

### 4.2. In Vitro Studies

#### 4.2.1. Inhibition of Hcov-OC43 Human Coronavirus

For in-vitro studies, test compounds were prepared in 20-mM solutions and sent to Institute for Viral Research (USU) for analysis. Compounds were serially diluted using eight half-log dilutions in test medium (MEM supplemented with 5% FBS and 50 μg/mL gentamicin) so that the starting (high) test concentration was 100 μM. Each dilution was added to 5 wells of a 96-well plate with 80–100% confluent RD cells. Three wells of each dilution were infected with virus, and two wells remained uninfected as toxicity controls. Six wells were infected and untreated as virus controls, and six wells were uninfected and untreated as cell controls. hCoV-OC43 virus was prepared to achieve the lowest possible multiplicity of infection (MOI) that would yield >80% cytopathic effect (CPE) within 5 days. M128533 was tested in parallel as a positive control. Plates were incubated at 37 ± 2 °C, 5% CO_2_. 

On day 5 post-infection, once untreated virus control wells reached maximum CPE, plates were stained with neutral red dye for approximately 2 h (±15 min). Supernatant dye was removed and wells rinsed with PBS, and the incorporated dye was extracted in 50:50 Sorensen citrate buffer/ethanol for >30 min and the optical density was read on a spectrophotometer at 540 nm. Optical densities were converted to percent of cell controls and normalized to the virus control, then the concentration of test compound required to inhibit CPE by 50% (EC50) was calculated by regression analysis. The concentration of compound that would cause 50% cell death in the absence of virus was similarly calculated (CC50). The selective index (SI) is the CC50 divided by EC50.

#### 4.2.2. Kinase Inhibition Assay

The HotSpot kinase profiling assay platform (Reaction Biology Corporation) was used to kinase inhibitory properties of FBL-03G. The detailed protocol for the assay was previously described by Anastassiadis et al., 2011 [32]. In brief, for each reaction, kinase and substrate were mixed in a buffer containing 20 mM HEPES (pH 7.5), 10 mM MgCl_2_, 1 mM EGTA, 0.02% Brij35, 0.02 mg/mL BSA, 0.1 mM Na_3_VO_4_, 2 mM DTT, and 1% DMSO. Compounds were then added to each reaction mixture. After a 20-min incubation, ATP (Sigma-Aldrich, Saint Louis, MO, USA) and [g-33P] ATP (PerkinElmer, Waltham, MA, USA) were added at a final total concentration of 10 mM. Reactions were carried out at room temperature for 2 h and spotted onto P81 ion exchange cellulose chromatography paper (Whatman). Filter paper was washed in 0.75% phosphoric acid to remove unincorporated ATP. The percent remaining kinase activity relative to a vehicle-containing (DMSO) kinase reaction was calculated for each kinase/inhibitor pair. Outliers were identified and removed. IC_50_ values were calculated using GraphPad Prism 5 (GraphPad Software, San Diego, CA, USA).

#### 4.2.3. Proinflammatory Cytokine Inhibition

The Human Cytokine/Chemokine Magnetic bead panel from Millipore (Catalog # HCYTOMAG-60K) was used in this study according manufacturer’s instructions. Monocyte/macrophage inhibition in human PBMC cells stimulated with LPS was analyzed. Cryopreserved human PBMC cells were seeded in 96 well plates at density of 5 × 10^4^ cells/well in 150 μL RPMI culture media. The plates were incubated for 1 h at 37 °C with 5% CO_2_. To the wells, 10 μL diluted compounds, Dexamethasone, and vehicle controls were added to appropriate wells according to the plate map. The plates were incubated at 37 °C with 5% CO_2_ for 1 h. To appropriate wells, 40 μL LPS (*E. coli*, 50 pg/mL) was added and plates further incubated at 37 °C, 5% CO_2_, for 24 h. The plates were centrifuged at 1000 rpm for 10 min and supernatant harvested. The supernatants were transferred to a clean plate and stored at -80 °C until ready to analyze on the Luminex and cytokine (IL-1β, IL-6, IL-8, MIP-1α and TNFα) levels measured. Levels of cytokine induction were interpolated off a standard curve using a 5-point non-linear regression analysis. The interpolated data was normalized to vehicle control and analyzed to generate IC_50_ values.

### 4.3. Smart Nanoparticles Studies

Smart nanoparticles discussed in this study were made using: gold (III) chloride trihydrate (HAuCl_4_.3H_2_O), THPC- tetrakis (hydroxymethyl) phosphnium chloride (80% solution in water), sodium hydroxide, dimethyl sulfoxide (DMSO), and HPLC grade water procured from Sigma Aldrich and used without further purifications. Mono-functional mPEG-thiol (Mw: 2000 Da) and hetero bi-functional anime-PEG-thiol (Mw: 3,400 Da) and carboxymethyl-PEG-thiol (Mw: 2000 Da) from Laysan Bio Inc were used for the PEGylation of as-prepared gold nanoparticles. Fluorophore, Alexa Fluor 647 (AF647) carboxylic acid, succinimidyl ester was purchased from Invitrogen (Carlsbad, CA, USA). For purification of nanoparticle, cellulose dialysis membrane (12–14 kDa) and microfuge membrane filters (NANOSEP 100K OMEGA) were procured from Sigma Aldrich (Saint Louis, MO, USA) and Pall Corporation (Westborough, MA, USA), respectively.

THPC capped nanoparticles were synthesized by controlled reduction of auric (III) chloride as reported previously by our group. Briefly, 0.5 mL of freshly prepared 1 M NaOH was added to 45 mL of HPLC grade water followed by addition of 1 mL of THPC solution (prepared by adding 12 μL of 80% THPC in water to 1 mL of HPLC water). The solution was stirred vigorously at room temperature. After 5 min of stirring, 2 mL of 25 mM HAuCl_4_ was added directly into the stirring mixture. A rapid change of color of the stirring mixture (from yellow to dark brown) indicated the reduction of HAuCl_4_ and formation of stabilized nanoparticles. The stirring was continued for another 15 min. For grafting the PEG/functionalized PEG on the surface of as-prepared nanoparticles, ligand exchange method was used by adding 2 mL of 3.75 mg/mL mPEG-thiol, 2 mL of carboxymethyl-PEG-thiol, and 4 mL of amine-PEG-thiol to the 45 mL of as-prepared nanoparticles. The reaction mixture was gently stirred overnight at room temperature. The PEGylated nanoparticles were purified using dialysis against distilled water for 24 h using a cellulose membrane with a cut-off size of 12–14 kDa. The dialyzed nanoparticles were sterile filtered using a 0.45-μm syringe filter and lyophilized to obtain dried PEGylated gold nanoparticles (pGNPs). pGNPs were stored at 4 °C for future use. The fluorophore AF647 was conjugated to the amine groups of the PEG on the surface of the pGNPs. Briefly, 2 mg of pGNPs were dispersed in 1 mL of carbonate buffer (0.1 M, pH 8.8) followed by addition of 5 μL of 10 mg/mL AF647 succinimidyl ester in DMSO. The reaction mixture was gently stirred for 2 h at room temperature. The unbound or free AF647 was separated from the AF647 conjugated pGNPs using 100 kDa centrifugal spin filters. The purified AF647 conjugated pGNPs (AF647-pGNPs) were washed three times with HPLC grade water to ensure complete removal of free fluorophore. For active targeting to lung lesions, RGD peptide was conjugated to carboxyl groups of the PEGylated pGNPs via carbodiimide chemistry, and the purified nanoparticles were lyophilized for future use.

All in vivo experiments performed in this study were approved by the Animal Care and Use Committee of the Dana-Farber Cancer Institute (protocol number 17007). Transgenic mouse models bearing single-nodule lung lesions were generated as described in recent work [33]. RGD targeted pGNPs were administered to cohort A mice via INH, while the same concentration of RGD-pGNPs was administered to cohort B mice via i.v. The nanoparticles distribution was measured via fluorescence imaging and ex-vivo electron microcopy methods. The optical fluorescence imaging was done using Maestro GNIR FLEX fluorescence imaging system (CRI) using a 633-nm excitation filter. Mice were dissected and entire lungs were imaged 24 h post nanoparticles administration. The images were acquired with the same acquisition time of 88 ms for all the experiments. A quantitative estimation of the fluorescence intensity was done using the Maestro software and Image J.

## Figures and Tables

**Table 1:** Antiviral activity of caflanone against human coronavirus (-OC43) and viral/host factors *.

Bioactivity	EC_50_/IC_50_ (µM)
hCov-OC43 beta virus	0.42
ABL-2	0.27
AXL	<5.0
Cathepsin L	3.28
IL-1β	2.4
IL-6	9.1
IL-8	9.9
Mip-1α	8.9
TNF-α	8.7
CK2a2	0.038
JAK2	1.85
MNK2	0.549
PI4Kiiiβ	0.136

* The hCov-OC43 virus was cultured in RD (Rhabdomyosarcoma) cells while the Hotspot kinase profiling assay was used to assess kinase inhibition. Proinflammatory cytokine inhibition was measured in human PBMC (Peripheral blood mononuclear cells) cells stimulated with LPS (Liposaccharide). ABL2 (Abelson Murine Leukemia Viral Oncogene Homolog 2), AXL (AXL Receptor Tyrosine Kinase), IL-1β (Interleukin 1β), IL-6 (Interleukin 6), IL-8 (Interleukin 8), Mip-1α (macrophage inflammatory protein 1α), TNF-α (Tumor Necrosis Factor-α), CK2a2 (Casein kinase 2), JAK2 (Janus kinase 2), MNK2 (Mitogen-activated protein kinase signal-integrating kinase), PI4Kiiiβ (Phosphatidylinositol 4-kinase III beta).
